# Understandable, individualised information about risk is essential to help patients make fully informed decisions about breast cancer treatment

**DOI:** 10.1136/bmjonc-2025-000762

**Published:** 2025-03-21

**Authors:** Shelley Potter

**Affiliations:** 1 Translational Health Sciences, University of Bristol Medical School, Bristol, UK; 2 Bristol Breast Cancer Centre, NHS North Bristol NHS Trust, Bristol, England, UK

**Keywords:** Breast cancer (female)

 Breast cancer treatment has become increasingly complex and so too have the decisions patients face regarding their care.

Patients with newly diagnosed early breast cancer need to make decisions about breast and axillary surgery which may include complex oncoplastic and reconstructive options and adjuvant treatments with variable combinations of chemotherapy, radiotherapy, endocrine therapy and targeted treatments offered depending on the tumour stage and subtype.^1^ Increasing use of neoadjuvant systemic therapies introduces additional decisions about treatment sequencing and the optimal use of response-adapted locoregional and adjuvant systemic treatment strategies. In addition, genetic testing is offered to increasing numbers of patients to identify germline mutations not only to inform surgical decision-making, but also to identify individuals who may benefit from systemic therapy with PARP (polymeric adenosine diphosphate ribose polymerase) inhibitors.

Improvements in treatments together with earlier detection via breast screening programmes have significantly improved long-term breast cancer survival.^2^ However, incremental improvements in oncological outcomes have traditionally been achieved by the addition of new or better treatments. This has led to the perception among both patients and clinicians that ‘more’ breast cancer treatment is ‘better’ at reducing the risk of breast cancer recurrence. While this is true for some patients, especially those with high-risk disease, as our understanding of breast cancer tumour biology has improved, it is becoming clear that not all patients need or will benefit from all the treatments that they may have traditionally received. Indeed, while these patients may derive no oncological benefit from these treatments, most, if not all, will experience treatment-related complications, side-effects and morbidities which may impact their long-term well-being and quality of life.

Discussing the reduction or omission of treatments, even if those treatments may have little or no benefit, however, can be challenging for professionals.^3^ Clinicians need to be able to accurately communicate the absolute benefits and risks of treatments informed by the individual’s tumour characteristics and explore the patient’s values and preferences to reach a shared decision. Key to these discussions is the acknowledgement of uncertainty as clinicians need to base discussions on estimations of risk and benefit.

Gene expression profiling (GEP) tests such as Oncotype DX and Prosigna use multigene signatures from the patient’s own cancer to provide personalised information about the risk of breast cancer recurrence with a ‘recurrence score’. These tests can inform chemotherapy decision-making with a low recurrence score indicating minimal chemotherapy benefit and supporting safe omission of treatment and a high score identifying patients at higher risk of recurrence who would potentially benefit from treatment. Patients offered these tests, however, need to understand the rationale for testing and the meaning of the results, and industry-based information resources are complex and may have limited value for patients.

In the IMPARTER4 study,^4^ Fallowfield and colleagues evaluated the impact of a short 8-min film explaining GEP tests that was developed in collaboration with clinicians and patients on patients’ understanding of the tests and their results. A total of 251 patients with early breast cancer having GEP tests at 18 UK centres were randomised to receive standard information about the test with or without the addition of the video. Patients randomised to receive the video showed significantly increased knowledge scores compared with those receiving standard information alone. In addition, clinicians reported consultations with patients who had received the video were shorter and less complex, and patients reported valuing the film. While the researchers did not evaluate the content of the consultations themselves, this trial provides high-quality evidence that simple, patient-friendly information films about GEP tests had a positive impact on informed decision-making for chemotherapy. It could be hypothesised that developing similar resources to support informed decision-making for other breast cancer treatment decisions may also have utility, but lack of appropriate risk-stratification tools may limit their value. Currently, Oncotype and Prosigna are only validated for chemotherapy, although studies to evaluate their utility in selecting patients for radiotherapy omission are currently underway.^5 6^ Other genomic profiles to select patients for omission of radiotherapy (POLAR)^7^ and to determine the benefit of extended endocrine therapy (Breast Cancer Index)^8^ have been developed but are not yet standard of care.

Patients with breast cancer want high-quality, balanced, personalised information to support breast cancer decision-making,^9^ particularly when the benefits of treatments may be small, but this information needs to be acceptable and understandable if these decisions are truly informed. In the digital era, traditional written information alone is no longer sufficient, and more patient-friendly resources, co-developed with patients, are needed. As decisions become more complex and nuanced, however, the role of the clinician in helping patients navigate these challenges cannot be overstated. Clinicians now, more than ever, need to be able to communicate personalised information about potential risks and benefits and to work with patients to understand their individual preferences to support meaningful shared decision making.

## Data Availability

Data sharing not applicable as no datasets generated and/or analysed for this study.
